# Effects of cosmetic ingredients on growth and virulence factor expression in *Staphylococcus aureus*: a comparison between culture medium and *in vitro* skin model medium

**DOI:** 10.3934/microbiol.2025002

**Published:** 2024-12-24

**Authors:** Yuya Uehara, Yuko Shimamura, Chika Takemura, Shiori Suzuki, Shuichi Masuda

**Affiliations:** School of Food and Nutritional Sciences, University of Shizuoka, 52-1 Yada, Suruga-ku, Shizuoka 422-8526, Japan

**Keywords:** *in vitro* skin model, *Staphylococcus aureus*, cosmetic ingredients, virulence factor, staphylococcal enterotoxin A

## Abstract

The effect of cosmetic ingredients on growth and virulence factor expression in *Staphylococcus aureus* may vary between culture medium and skin. Researchers have used an *in vitro* skin model with human heel callus to assess bacterial survival and growth on the stratum corneum of the epidermis. Here, we reconstituted a skin model using keratin as a base (instead of callus) and compared it with brain heart infusion (BHI) medium. We investigated the effects of five cosmetic ingredients (macadamia nut oil, sodium myristoyl methyl taurate, methyl *p*-hydroxybenzoate, 2-phenoxyethanol, and zinc oxide) on growth and virulence factor expression in *S. aureus*. Interestingly, the survival pattern of *S*. *aureus* in our skin model was similar to that reported in models using callus. Upon the addition of cosmetic ingredients to BHI or skin model medium, the sensitivity of *S. aureus* to these cosmetic ingredients differed between the two media. Notably, after adding the two tested cosmetic ingredients, the expression level of staphylococcal enterotoxin A in *S. aureus* reduced significantly in skin model medium compared with that in the BHI medium. Additionally, the expression levels of other *S. aureus* virulence factors (RNAIII, *icaA*, and *hlb*) differed between the two media. These findings suggest that our skin model is a valuable tool for evaluating the effects of cosmetic ingredients on growth and virulence factor expression in *S. aureus*.

## 1. Introduction

The skin is composed of several layers, including the dermis and epidermis, and acts as a barrier against environmental and pathogenic threats. Within the densely populated microbial ecosystem of the skin, bacteria, fungi, and viruses interact with each other and the immune system to maintain skin health and homeostasis. *Staphylococcus aureus*, a normal skin inhabitant, can occasionally become pathogenic. It is considered the most pathogenic *Staphylococcus* species and produces an exotoxin known as staphylococcal enterotoxin A (SEA) and pathogenic factors, causing food poisoning and atopic dermatitis [1],[2]. The expression of several virulence factors in *S. aureus* is controlled by the accessory gene regulator (*agr*) quorum sensing system [3]. For the *agr* system, a cell density–dependent gene regulation mechanism is employed, and a signal molecule is recognized, known as an auto inducer peptide. The *agr* regulatory system comprises four protein genes (*agr*BDCA) and regulatory RNAs (small RNAs), namely, RNAII and RNAIII, and controls the expression of several virulence factor genes, including *hlb* (β-hemolysin) and *icaA* (biofilm formation) [4]. Conversely, researchers found that SEA expression remains unaffected by the *agr* system [5].

*S. epidermidis*, a member of the same genus as *S. aureus*, degrades triglycerides in sebum into fatty acids (acidic) and glycerol, thereby maintaining a slightly acidic environment of the skin. In healthy skin, *S. epidermidis* is considerably more prevalent than *S. aureus* [6]. Conversely, the proportion of *S. aureus* is higher under alkaline environment of the skin, as observed in the skin of atopic patients. *S. aureus* and *S. epidermidis* maintain host health by controlling growth through crosstalk of the skin via the quorum sensing system [7].

Cosmetics have been reported to inhibit survival and biofilm formation in *S. aureus* [8]. In a study, acrylamide increased SEA production and biofilm formation in *S. aureus* [9], suggesting that chemicals affect the pathogenicity of *S. aureus*. As cosmetic ingredients are directly applied to the skin, they may alter virulence factor expression in *S. aureus*. Toxicity evaluations of pathogenic bacteria, including *S. aureus*, are usually conducted under culture conditions. However, owing to significant differences between *in vivo* and culture medium components, we need models that better mimic living conditions for toxicity assessments. Unfortunately, only a few model systems have been established for studying skin-resident bacterial behavior *in vitro*. Under normal skin conditions, bacteria primarily reside in the upper stratum corneum, utilizing nutrients derived from cross-linked proteins and lipids [10]. Although an *in vitro* system using human callus as a substrate [11] has been reported to mimic the human stratum corneum, challenges remain in terms of reproducibility due to individual differences.

**Figure 1.**
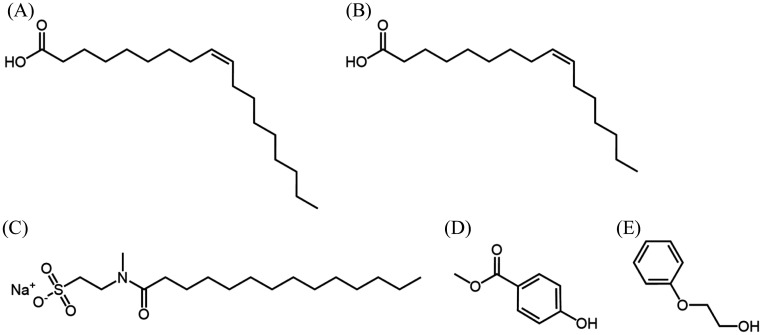
Structure of the cosmetic ingredients used in this study (except ZnO). (A) Oleic acid, (B) palmitoleic acid ((A) and (B) are the main component of macadamia nut oil (MO)), (C) sodium myristoyl methyl taurate (SMMT), (D) methyl *p*-hydroxybenzoate (MP), and (E) 2-phenoxyethanol (PE).

Herein, we developed a novel skin model using keratin as a base instead of human callus. The main source of nutrients for skin microorganisms are sweat and sebum [12]. Therefore, the skin model was based on keratin, along with the components of sweat and sebum. To assess *S. aureus* and *S. epidermidis* cocultures, we employed ethidium monoazide (EMA) treatment–dependent PCR (EMA-PCR) [13]. We compared the effects of five cosmetic ingredients (macadamia nut oil (MO), sodium myristoyl methyl taurate (SMMT), methyl *p*-hydroxybenzoate (MP), 2-phenoxyethanol (PE), and zinc oxide (ZnO)) on growth and virulence factor expression in *S. aureus* between our newly constructed *in vitro* model medium and brain heart infusion (BHI) medium. MO, primarily composed of oleic acid (40%–51%) and palmitoleic acid (24%–36%) [14] (Figure 1A,B), reportedly lacks antimicrobial activity against *S. aureus* [15]. SMMT (Figure 1C) is a sodium salt of the condensation product of myristic acid and N-methyl taurine, serving as an anionic surfactant, which is classified as an acyl methyl taurate of taurine surfactants [16]. MP (Figure 1D), the methyl ester of 4-hydroxybenzoic acid, is commonly used as a preservative due to its broad antibacterial spectrum [17]. Furthermore, PE (Figure 1E) is widely employed as an antiseptic and antibacterial agent in cosmetics and inhibits *S. aureus* growth at high concentrations [18]. ZnO, an inorganic ultraviolet scattering agent [19], reportedly inhibits *S. aureus* growth [20]. Owing to their versatility and widespread use, we evaluated the effects of these cosmetic ingredients using our *in vitro* skin model medium.

## 2. Materials and methods

### 2.1. Materials

The cosmetic ingredients were either dissolved or suspended in sterile water. Their concentrations were determined based on common formulation ratios used in cosmetics: 10% for MO [21]; 1% for SMMT; 0.1% for MP and PE; and 0.5% for ZnO (Figure 1). These cosmetic ingredients were provided by KOSÉ Corporation (Tokyo, Japan).

### 2.2. Bacterial strain and culture conditions

We used two bacterial strains, namely, *S. aureus* C-29 (isolated from a human hand producing staphylococcal enterotoxin A) [22] and *S. epidermidis* NBRC100911. *S. epidermidis* was included solely to establish an *in vitro* skin model medium, which was compared with other callus models. Both *S. aureus* and *S. epidermidis* were inoculated into BHI broth and incubated at 37 °C under shaking conditions for 24 h. The resulting bacterial solution (30 µL) was then inoculated in 3 mL of BHI broth and incubated similarly. After centrifugation (11,600 × g, 3 min), the supernatant was removed, and the bacteria were washed with phosphate-buffered saline (PBS). This procedure was repeated, and a bacterial suspension was obtained.

### 2.3. Preparation of in vitro skin model medium

We dissolved the ingredients listed in Table 1 in MilliQ water to prepare three *in vitro* skin model solutions. The *in vitro* skin model solution was prepared using the artificial sweat method D (JIS0848) and artificial finger sebum solution (containing 0.03% urea, 0.09% lactic acid, 0.20% sodium pyrophosphate decahydrate, 0.37% sodium chloride, and 0.50% ethanol) according to Japanese Industrial Standards. To construct a simple simulated medium, Model 1 was prepared using only sweat components. Model 2 was prepared by adding sebum components to Model 1, and Model 3 was prepared by adding glucose and squalane to Model 2. Squalane was provided by KOSÉ Corporation, keratin (derived from wool) was purchased from Tokyo Chemical Industry Co., Ltd. (Tokyo, Japan), ethanol was obtained from Kanto Chemical Co., Inc. (Tokyo, Japan), and other reagents were procured from Fujifilm Wako Pure Chemical Corporation (Osaka, Japan). We added Bacto agar (Becton, Dickinson and Company, NJ, USA) to PBS to achieve a final concentration of 2%, autoclaved the mixture, and dispensed it into 24- or 48 well plates (1 mL aliquots) to prepare 2% agar medium. Finally, we added 0.1 mL of the *in vitro* skin model solution to the agar medium, establishing *in vitro* skin model medium.

**Table 1.** Composition of the *in vitro* skin model solution.

Ingredient name	Addition ratio (%)		
	Model 1	Model 2	Model 3
Disodium hydrogen phosphate dodecahydrate	0.20	0.20	0.20
Sodium chloride	0.20	0.37	0.37
Acetic acid	0.13	0.26	0.26
Urea	－	0.03	0.03
Lactic acid	－	0.09	0.09
Sodium pyrophosphate decahydrate	－	0.20	0.20
Ethanol	－	0.50	0.50
Glucose	－	－	0.20
Squalane	－	－	5.0
Keratin	2.0	2.0	2.0

### 2.4. Measurement of viable bacteria during a single culture

The *S. aureus* or *S. epidermidis* bacterial solution was added to *in vitro* skin model medium to achieve initial bacterial counts of 10^3^, 10^5^, and 10^7^ CFU/well, followed by incubation at 37 °C. After 0, 1, and 4 days of incubation, 1 mL of PBS was added to each well to recover the bacterial solution. The recovered bacterial solution was appropriately diluted, and 100 µL of the diluted solution was plated onto mannitol salt medium and incubated at 37 °C for 48 h, after which the viable bacterial count was determined.

### 2.5. Measurement of viable bacteria during coculture using EMA-PCR

For EMA treatment, the *S. aureus* or *S. epidermidis* bacterial solution was added to *in vitro* skin model medium to achieve an initial bacterial count of 10^7^ CFU/well. The samples were then subjected to EMA-PCR using Viable Bacteria Selection Kit (gram-positive, TaKaRa Bio Inc., Shiga, Japan), following the manufacturer's instructions. For EMA modification, the mixture was left on ice for 5 min and then irradiated with light for 5 min twice, followed by a final 15 min irradiation. After heat treatment at 95 °C for 5 min, genomic DNA was extracted from both EMA-treated and non-EMA-treated bacterial solutions using QIAamp DNA Mini Kit (Qiagen, Netherlands). Real-time PCR was performed using a Thermal Cycler Dice® Real Time System II (TaKaRa Bio Inc.) and a PrimeScript RT Reagent Kit (TaKaRa Bio Inc.) according to the manufacturer's instructions. The primers used targeted the 16S rRNA gene of each bacterium, and a universal primer for the 16S rRNA gene was used for DNA amount correction (Table 2).

**Table 2.** Primers and their sequences.

Primer	Gene	Sequence (5′–3′)
338F	Universal 16S rRNA	ACTCCTACGGGAGGCAGCAG
518R		ATTACCGCGGCTGCTGG
SA-F	*S. aureus*	GATCAATTTATGGCTAGACG
SA-R		CGAAGGTTCTGTAGAAGTATGA
SE-F	*S. epidermidis*	CCATTCTGGACCGTTTAGTGGTT
SE-R		TTTGATGCGTGAGATACTTCTTCGT

### 2.6. Comparison of in vitro skin model medium and BHI medium

#### 2.6.1. Effect of cosmetic ingredients on the viable *S. aureus* count

The *S. aureus* bacterial solution was added to *in vitro* skin model medium to achieve an initial bacterial count of 10^7^ CFU/well. Cosmetic ingredients, including MP (0.1%), PE (0.1%), ZnO (0.5%), SMMT (1%), and MO (10%), were added, and the mixture was incubated at 37 °C for 24 h. Sterile water was used as a control. After incubation, 1 mL of PBS was added to each well to recover the bacterial solution. The recovered bacterial solutions were diluted, and 100 µL of the diluted solution was plated onto mannitol salt medium and incubated at 37 °C for 48 h, followed by bacterial counting. For comparison of *in vitro* skin model medium and BHI medium, the same evaluation was performed using BHI medium. The *S. aureus* bacterial solution was added to 1 mL of BHI medium to achieve an initial bacterial count of 10^7^ CFU/well. Then, MP (0.1%), PE (0.1%), ZnO (0.5%), SMMT (1%), and MO (10%) were added, and the mixture was incubated at 37°C for 24 h. Sterile water was used as a control. After incubation, the absorbance at 660 nm (OD_660_) was measured using a plate reader (FlexStation, Molecular Device, CA, USA). Bacterial counts in BHI broth were converted from OD_660_ values. The growth of *S. aureus* in the BHI medium was monitored, and the bacterial count (CFU/mL) at OD_660_ was obtained at various time points (0, 3, 6, 9, 24, 30, and 48 h). The following Eq 1 was obtained, where y represents the OD_660_ value and x represents the number of bacteria.

[y=0.291ln(x)−4.8034] (1)

#### 2.6.2. Effect of cosmetic ingredients on the expression of virulence factors in *S. aureus*

The *S. aureus* bacterial suspension was added to achieve an initial bacterial count of 10^7^ CFU/well. It was inoculated into *in vitro* skin model medium and 1 mL of BHI medium. Cosmetic ingredients were added at the same concentrations as described in Section 2.6.1, and the mixture was incubated at 37 °C for 6 h. After incubation, 1 mL of PBS was added to *in vitro* skin model medium to recover the bacterial solution. The recovered bacterial solution and BHI culture medium were centrifuged (14,000 × g, 1 min) to collect the bacterial cells. Then, RNA was extracted using RiboPure-Bacteria (Invitrogen, CA, USA). The total RNA concentration was measured using K2800 Nucleic Acid Analyzer (Beijing Kaiao Technology Development Co., Ltd., Beijing, China), and the total RNA content of each sample was adjusted to 500 ng using RNase-free distilled water. cDNA was synthesized via reverse transcription (RT) using PrimeScript RT Reagent Kit (TaKaRa Bio Inc.). Real-time RT–PCR was performed using PrimeScript RT Reagent Kit (TaKaRa Bio Inc.) and real-time PCR device (Thermal Cycler Dice® Real Time System II; TaKaRa Bio Inc.) according to the manufacturer's instructions. The 16S rRNA gene served as an internal standard to correct for mRNA levels between samples. The primer sequences are listed in Table 3. Heatmaps were generated using Heatmapper (http://www.heatmapper.ca/ accessed on May 3, 2024) [23].

**Table 3.** Primers and their sequences.

Primer	Gene	Sequence (5′–3′)
16S rRNA-F	16S rRNA	CGTGCTACAATGGACAATACAAA
16S rRNA-R		ATCTACGATTACTAGCGATTCCA
sea-F	*sea*	GATCAATTTATGGCTAGACG
sea-R		CGAAGGTTCTGTAGAAGTATGA
RNAIII-F	RNAIII	CGATGTTGTTTACGATAGCTT
RNAIII-R		CCATCCCAACTTAATAACCA
icaA-F	*icaA*	AGTTGTCGACGTTGGCTA
icaA-R		CCAAAGACCTCCCAATGT
hlb-F	*hlb*	GCGGTTGTGGATTCGATAAT
hlb-R		CAGCACCACAACGTGAATCT

### 2.7. Statistical analysis

Experimental results are presented as means ± standard deviation. Statistical analysis was performed using Student *t*-test and two-way analysis of variance with Sidak's multiple comparisons test via Microsoft Excel 2016 (Microsoft, Redmond, WA, USA), with a significance level set at *p* < 0.05.

## 3. Results

### 3.1. Construction of in vitro skin model medium

#### 3.1.1. Validation of *in vitro* skin model medium for the culture of *S. aureus* and *S. epidermidis*

**Figure 2.**
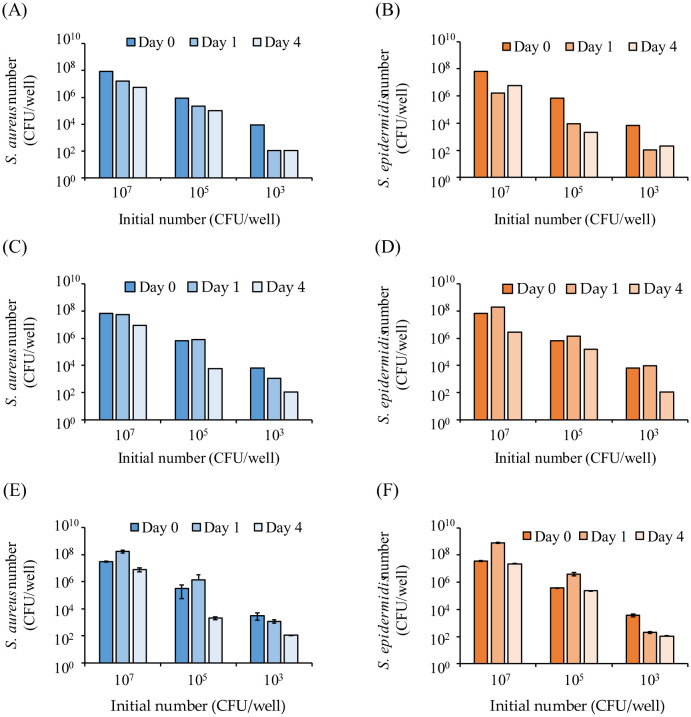
Viable counts of *Staphylococcus aureus* and *S. epidermidis* incubated in the *in vitro* skin model medium with different compositions. Viable counts of (A) *S. aureus* and (B) *S. epidermidis* based on Model 1 (n = 2). Viable counts of (C) *S. aureus* and (D) *S. epidermidis* based on Model 2 (n = 2). Viable counts of (E) *S. aureus* and (F) *S. epidermidis* based on Model 3 (n = 3). The *S. aureus* and *S. epidermidis* bacterial solutions were added to the *in vitro* skin model medium at initial bacterial counts of 10^3^, 10^5^, and 10^7^ CFU/well, and the bacterial counts were measured after incubation for 0, 1, and 4 days.

We prepared three *in vitro* skin model media (Models 1, 2, and 3) with different compositions and examined the viable counts of *S. aureus* and *S. epidermidis*. In Models 1 and 2, the bacterial counts decreased over time for *S. aureus*, regardless of the initial bacterial count (Figure 2A–D). Conversely, in Model 3, when the initial bacterial count was ≥10^5^ CFU/well, the bacterial count increased on the first day and decreased on the fourth day (Figure 2E,F). This growth pattern matched that of the previously reported callus model [11]. Consequently, in subsequent experiments, we used Model 3 as the *in vitro* skin model medium to evaluate changes in viable bacterial counts during coculturing of *S. aureus* and *S. epidermidis*.

#### 3.1.2. Validation of *in vitro* skin model medium for the coculture of *S. aureus* and *S. epidermidis*

**Figure 3.**
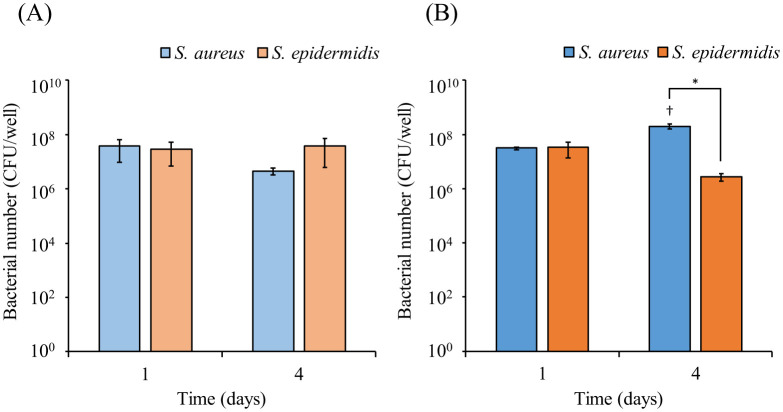
The bacterial counts of *Staphylococcus aureus* and *S. epidermidis* after 1 and 4 days of culture in the *in vitro* skin model medium (Model 3). Measurements in (A) monoculture and (B) coculture. Bacterial counts were assessed using EMA treatment, followed by genomic DNA extraction and real-time PCR. The values represent the mean ± standard deviation (SD) of three independent experiments. Significance levels: *p < 0.05 compared with *S. aureus* after 4 days, †*p* < 0.05 compared with *S. aureus* after 1 day (two-way ANOVA with Sidak's multiple comparisons test).

We evaluated the bacterial counts of cocultured *S. aureus* and *S. epidermidis* in the *in vitro* skin model medium using EMA-PCR. When *S. aureus* or *S. epidermidis* were cultured alone in the *in vitro* skin model medium, only the viable count of *S. aureus* tended to decrease on day 4 (Figure 3A). However, when the strains were cocultured, the viable count of *S. aureus* increased, whereas that of *S. epidermidis* decreased on day 4 (Figure 3B).

### 3.2. Comparison of the viable counts of S. aureus between in vitro skin model medium and BHI medium upon the addition of cosmetic ingredients

**Figure 4.**
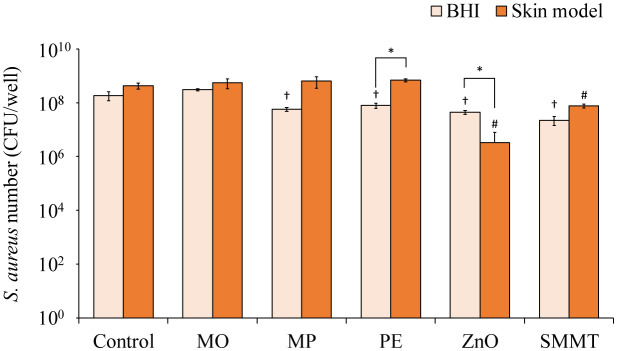
Comparison of *Staphylococcus aureus* bacterial counts in the *in vitro* skin model medium and BHI medium upon the addition of cosmetic ingredients. *S. aureus* was inoculated into the *in vitro* skin model medium and BHI medium and incubated at 37°C for 24 h. MO: 10% macadamia nut oil; MP: 0.1% methyl *p*-hydroxybenzoate; PE: 0.1% 2-phenoxyethanol; ZnO: 0.5% zinc oxide; and SMMT: 1% sodium myristoyl methyl taurate. Sterile water was used as a control. * *p* < 0.05, †*p* < 0.05 compared with the control of BHI medium, and # *p* < 0.05 compared with the control of skin model medium (two-way ANOVA with Sidak's multiple comparisons test).

Next, we assessed the effects of cosmetic ingredients on *S. aureus* growth using *in vitro* skin model medium and BHI medium. In the *in vitro* skin model medium, no significant change in the number of viable bacteria was observed with the addition of MO, MP, and PE (Figure 4). However, the addition of ZnO and SMMT in the *in vitro* skin model medium significantly decreased the number of viable cells (Figure 4). In the BHI medium, all cosmetic ingredients, except for MO, significantly reduced the viable bacterial count. Furthermore, the number of viable *S. aureus* cells increased in the *in vitro* skin model medium with the addition of MP and PE, whereas in the BHI medium, the addition of ZnO resulted in a higher reduction of viable cells (Figure 4).

### 3.3. Comparison of the expression of S. aureus virulence factors upon the addition of cosmetic ingredients between in vitro skin model medium and BHI medium

**Figure 5.**
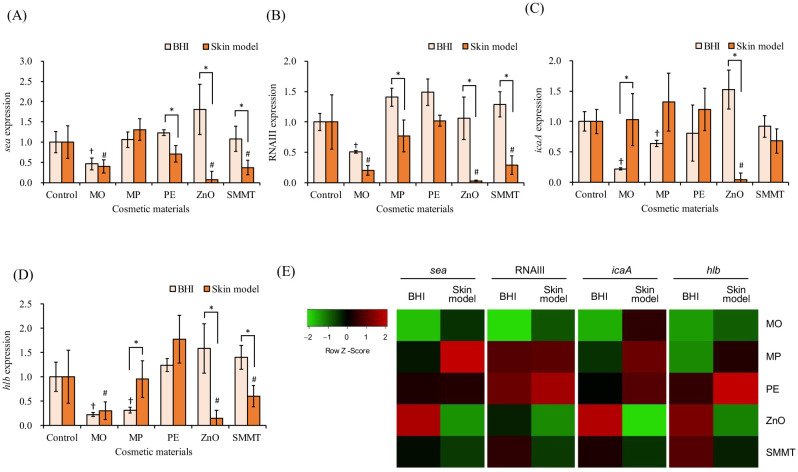
Comparison of the effects of five cosmetic ingredients on the expression of *S. aureus* virulence factor genes between *in vitro* skin model medium and BHI medium. (A) staphylococcal enterotoxin A (*sea*). (B) RNAIII. (C) β-hemolysin gene (*hlb*). (D) biofilm formation gene (*icaA*). (E) Heatmaps showing changes in gene expression levels in the BHI medium and *in vitro* skin model medium. Differential expression of mRNA is shown by the intensity of red (upregulation) versus green (downregulation). *Staphylococcus aureus* was inoculated into the *in vitro* skin model medium and BHI medium containing cosmetic ingredients and incubated at 37 °C for 6 h. MO: 10% macadamia nut oil; MP: 0.1% methyl *p*-hydroxybenzoate; PE: 0.1% 2-phenoxyethanol; ZnO: 0.5% zinc oxide; and SMMT: 1% sodium myristoyl methyl taurate. Sterile water was used as a control. * *p* < 0.05, †*p* < 0.05 compared with the control of BHI medium, and #*p* < 0.05 compared with the control of skin model medium (student *t*-test).

Regarding gene expression levels, in the *in vitro* skin model medium, the addition of MO, ZnO, and SMMT significantly decreased the expression levels of *sea* (Figure 5A) and RNAIII (Figure 5B), but no significant changes were observed with the addition of MP and PE. The expression levels of *sea* (Figure 5A) and RNAIII (Figure 5B) in the BHI medium decreased significantly by the addition of MO and ZnO, whereas other cosmetic ingredients did not significantly alter these levels. In the *in vitro* skin model medium, the addition of PE, ZnO, and SMMT significantly decreased *sea* expression level, whereas that of MP, ZnO, and SMMT significantly lowered RNAIII expression level compared with those in the BHI medium.

Furthermore, *icaA* expression in the *in vitro* skin model medium was significantly decreased by ZnO, whereas no significant change was observed with the addition of other cosmetic ingredients (Figure 5C). In the BHI medium, MO and MP significantly reduced *icaA* expression, whereas other cosmetic ingredients did not significantly affect it (Figure 5C). Additionally, MO showed significantly lower expression of *icaA* in BHI medium than in the *in vitro* skin model medium, and ZnO exhibited significantly lower expression of *icaA* in the *in vitro* skin model medium than in BHI medium.

The expression level of *hlb* in the *in vitro* skin model medium was significantly reduced by MO, ZnO, and SMMT, whereas no change was observed with the addition of MP and PE (Figure 5D). In contrast, in the BHI medium, MO and MP significantly decreased *hlb* expression, whereas other cosmetic ingredients did not significantly alter it (Figure 5D). Additionally, MO exhibited significantly lower *hlb* expression in the BHI medium than in the *in vitro* skin model medium, and ZnO showed significantly lower expression in the *in vitro* skin model medium than in the BHI medium.

The heatmap illustrates a comparison of cosmetic ingredients–induced expression of virulence factor genes (*sea*, RNAIII, *icaA*, and *hlb*) in *S. aureus* between the BHI medium and *in vitro* skin model medium (Figure 5E). After the addition of MO, MP, and PE, the expression levels of all virulence factor genes were lower in the BHI medium than in the *in vitro* skin model medium. Conversely, after the addition of ZnO and SMMT, the expression levels of all virulence factor genes were lower in the *in vitro* skin model medium than in the BHI medium.

## 4. Discussion

Indigenous bacteria, including *S. aureus*, naturally inhabit the skin (dermal indigenous bacteria) and substantially influence skin conditions. Although it is believed that the properties of these indigenous bacteria change with cosmetic product usage, direct evaluation of this hypothesis on actual human skin is challenging. Consequently, researchers have turned to human reconstructed and *ex vivo* skin models [24]–[30]. However, these models are resource-intensive and costly to evaluate. An alternative approach is to use human callus as a substitute for the stratum corneum, supporting the growth of both indigenous skin bacteria and pathogenic bacteria [11]. Nevertheless, collecting human callus presents difficulties, and individual variations may affect experimental reproducibility. In this study, we investigated an *in vitro* skin model medium using keratin—a component of the stratum corneum—as an alternative to callus.

Three model solutions, designed to mimic skin components, were examined. In Model 3, when the initial bacterial count was ≥10^5^ CFU/well, both *S. aureus* and *S. epidermidis* counts increased on day 1 and decreased on day 4, showing a trend similar to that observed in the previously reported callus model [11] (Figure 1A,B). Interestingly, in the callus model, co-inoculation of *S. aureus* and *S. epidermidis* resulted in reduced *S. epidermidis* bacterial count compared with *S. aureus* inoculation alone [11]. Under stress-free growth conditions, such as in BHI medium, *S. aureus* grows faster than *S. epidermidis* [31]. In our preliminary experiments, *S. aureus* and *S. epidermidis* were co-cultured in BHI medium and *in vitro* skin model medium, and colonies were detected by the plate dilution method. Comparison of the two media on day 4 showed that the BHI medium was almost exclusively occupied by *S. aureus* colonies, whereas *in vitro* skin model medium was dominated by *S. aureus*, but also showed colonies of *S. epidermidis* (Figure S1). It was also reported that when *S. aureus* and *S. epidermidis* were co-cultured at pH 6, biofilms derived from both were formed in roughly equal amounts, whereas at pH 7, the majority of the biofilms were from *S. aureus* [31]. The *in vitro* skin model medium is based on the artificial sweat method D (pH 4.1) and is therefore slightly acidic, whereas the BHI medium is pH 7.4. In addition to the difference in nutrient source, the pH is thought to alter the proportion of *S. aureus* and *S. epidermidis* present. To evaluate this behavior, we decided to co-culture both strains in the *in vitro* skin model medium (Model 3) to determine the number of *S. aureus* and *S. epidermidis*. On mannitol salt medium, damaged *S. aureus* colonies are smaller (small colony variants) and are difficult to distinguish from *S. epidermidis* [32]. In addition, when *S. aureus* is predominant, the entire mannitol salt medium turns yellow, making it impossible to accurately detect *S. epidermidis*. Furthermore, cells that are alive but cannot be cultured are not detectable by culture methods [33]. Therefore, to determine the exact number of bacteria when *S. aureus* and *S. epidermidis* coexisted, the number of each was determined using EMA-qPCR. As expected, the bacterial count of *S. aureus* increased, whereas that of *S. epidermidis* decreased over the 4 day culture period (Figure 2), and *S. aureus* did not dominate as in the BHI medium (Figure S1). These results confirm that the *in vitro* skin model medium used in this study closely resembles the callus model. Notably, factors such as sex, age, lifestyle, and health status influence the composition of the skin microbiota [34]–[37]. Additionally, individual variations in skin thickness, sweating, and sebum production [38] contribute to differences in callus composition. Given its reproducibility and ease of preparation without using biological samples from humans, Model 3 was deemed the most suitable medium for the *in vitro* skin model and was used in subsequent experiments.

Next, we compared the effects of cosmetic ingredients on viable *S. aureus* counts between *in vitro* skin model medium and BHI medium. In the *in vitro* skin model medium, no significant changes in viable bacterial counts were observed with the addition of MO, MP, or PE (Figure 3). However, ZnO and SMMT significantly reduced the number of viable cells. In the BHI medium, all cosmetic ingredients, except for MO, significantly decreased viable bacterial counts (Figure 3). ZnO and myristic acid released from SMMT have been reported to inhibit *S. aureus* growth [39]–[41], which aligns with the current findings. MP, PE, and ZnO are commonly used in cosmetics as antiseptics and antibacterial agents [8],[42]. These cosmetic ingredients effectively inhibited *S. aureus* growth in the BHI medium in this study. Interestingly, MP and PE did not exhibit the same inhibitory effect on *S. aureus* growth in the *in vitro* skin model medium (Figure 3). The presence of keratin in the skin model medium, which can bind to *S. aureus* via surface proteins [43],[44], may have contributed to the discrepant outcomes observed for MP and PE between the two media.

In addition, the effects of cosmetic ingredients on the expression levels of virulence factors (*sea*, RNAIII, *icaA*, and *hlb*) in *S*. *aureus* were compared between *in vitro* skin model medium and BHI medium (Figure 4A–D). We have previously shown that suppressing the expression levels of *sea*, RNAIII, *icaA*, and *hlb* reduces the toxicity of extracellular membrane vesicles derived from *S. aureus*. Furthermore, membrane vesicles obtained under conditions that suppressed the expression of these four virulence factors significantly reduced the expression levels of inflammation-related genes in HaCaT cells [45] as well as allergy-related genes in a rat cell line derived from basophilic leukemia-2H3 cells [46]. Therefore, the expression levels of these four virulence factors were examined in this study. The heatmaps illustrate the comparison of cosmetic ingredients–induced expression of virulence factors in *S. aureus* between BHI medium and *in vitro* skin model medium (Figure 4E). The addition of MO significantly decreased the expression level of *sea* in both media, and that of ZnO and SMMT also significantly decreased the expression level of *SEA* in the *in vitro* skin model medium (Figure 4A). ZnO nanoparticles have been reported to reduce *sea* expression [47], and the current study reported a similar effect. Furthermore, the presence of organic acids such as acetic acid resulted in extremely low levels of *sea* mRNA [48]. Given that the skin model medium used in this study contained acetic acid and lactic acid, it is possible that these organic acids contributed to the differences in the effects of cosmetic ingredients on *sea* expression.

The expression level of RNAIII was significantly decreased by MO in both media as well as by ZnO and SMMT in the *in vitro* skin model medium (Figure 4B). Additionally, the expression level of *icaA*, which is involved in biofilm formation, was significantly reduced by ZnO in the *in vitro* skin model medium and by MO and MP in the BHI medium (Figure 4C). Biofilms, in which microorganisms adhere to surfaces and grow systematically, are composed of glycoproteins and extracellular DNA [49]. They can evade the action of antimicrobial agents and host immunity, leading to chronic inflammation [50]. Plant-derived essential oils with antimicrobial activity [51] and ZnO [52]–[56] have been reported to inhibit biofilm formation by suppressing the expression of RNAIII and *icaA*, and similar effects were observed in the *in vitro* skin model medium in the current study. However, MP and PE did not reduce *icaA* expression in the *in vitro* skin model medium, possibly due to the weakening of the bactericidal effect.

The expression level of *hlb* was significantly reduced by MO, ZnO, and SMMT in the *in vitro* skin model medium as well as by MO and MP in the BHI medium (Figure 4D). *hlb* reportedly plays a crucial role in the formation of *S. aureus* skin colonies [8]. In the skin model medium, bacterial growth was inhibited by ZnO and SMMT, corroborating the findings of previous studies [38]–[40]. Notably, *hlb* acts as an erythrocyte-lysing toxin [57] and damages human keratinocytes through sphingomyelinase activity [58]. Zn^2+^ inhibits sphingomyelinase activity in *Bacillus cereus* [59]. In the context of *in vitro* skin model cultures, MO and SMMT may suppress sphingomyelinase synthesis.

The expression of many virulence factors in *S. aureus* is regulated by the *agr* system via quorum sensing mechanisms [60]. It has been reported that the regulation of the *agr* regulatory system, including RNAIII and its downstream genes, varies between nutrient-poor and nutrient-rich environments [61]. Consequently, the expression of *agr* regulatory system genes in response to chemical additions may differ between the nutrient-poor *in vitro* skin model medium and nutrient-rich BHI medium. Given that actual skin exists in a nutrient-poor environment, the skin model used in the current study is valuable for evaluating the effects of cosmetic ingredients on growth and virulence factor expression in *S. aureus* on the skin.

## 5. Conclusions

In this study, we constructed an *in vitro* skin model medium using keratin, a component of the stratum corneum, instead of human callus. The survival patterns of *S. aureus* and *S. epidermidis* were similar to those reported in existing *in vitro* human callus models. By comparing the effects of five cosmetic ingredients on growth and virulence gene expression in *S. aureus*, we observed differences between the *in vitro* skin model medium and BHI medium. The susceptibility of *S. aureus* to cosmetic ingredients differed between the two media. The *in vitro* skin model medium constructed in this study may be used to evaluate the effects of cosmetic ingredients on *S. aureus* in an environment more similar to human skin. This is expected to enhance our understanding of growth dynamics and virulence factor expression in *S. aureus*.

## Use of AI tools declaration

The authors declare that they have not used artificial intelligence (AI) tools in the preparation of this article.


