# Supplementary material for: Effects of cosmetic ingredients on growth and virulence factor expression in Staphylococcus aureus: a comparison between culture medium and in vitro skin model medium
Source: AIMS Microbiol. 2024 Dec 24;11(1):22–39. doi: 10.3934/microbiol.2025002 (PMC11950679; doi:10.3934/microbiol.2025002)
Supplement: Supplementary file 1 [file microbiol-11-01-002-s001.pdf]

Microbiology

AIMS Microbiology, 11(1): 22–39.
DOI: 10.3934/microbiol.2025002
Received: 03 July 2024
Revised: 16 November 2024
Accepted: 17 December 2024
Published: 24 December 2024

https://www.aimspress.com/journal/microbiology

Research article

Effects of cosmetic ingredients on growth and virulence factor expression in *Staphylococcus aureus*: a comparison between culture medium and *in vitro* skin model medium

Yuya Uehara, Yuko Shimamura*, Chika Takemura, Shiori Suzuki and Shuichi Masuda*

School of Food and Nutritional Sciences, University of Shizuoka, 52-1 Yada, Suruga-ku, Shizuoka 422-8526, Japan

* **Correspondence:** Email: shimamura@u-shizuoka-ken.ac.jp; masudas@u-shizuoka-ken.ac.jp; Tel: +81542645528; Fax: +81542645528.

Supplementary

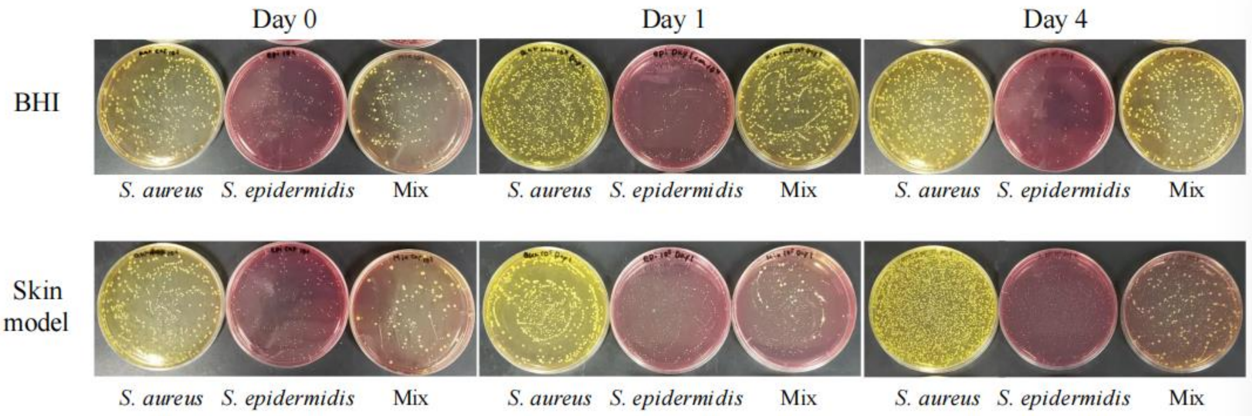

Figure S1. Colonies of *Staphylococcus aureus* and *Staphylococcus epidermidis* cultured in brain heart infusion (BHI) broth and in vitro skin model medium. *S. aureus*, *S. epidermidis*, and *S. aureus* and *S. epidermidis* (Mix) were inoculated into BHI broth and *in vitro* skin model medium with initial bacterial counts of 107 CFU/well each and incubated at 37 °C for 0, 1 and 4 days. At each time point, the inoculum was diluted as appropriate and spread on mannitol salt agar medium and incubated at 37 °C for 48 h.

AIMS Press

© 2025 the Author(s), licensee AIMS Press. This is an open access article distributed under the terms of the Creative Commons Attribution License (https://creativecommons.org/licenses/by/4.0)
